# γδ T cells in human colon adenocarcinomas comprise mainly Vδ1, Vδ2, and Vδ3 cells with distinct phenotype and function

**DOI:** 10.1007/s00262-024-03758-7

**Published:** 2024-07-02

**Authors:** William Rodin, Louis Szeponik, Tsvetanka Rangelova, Firaol Tamiru Kebede, Tobias Österlund, Patrik Sundström, Stephen Hogg, Yvonne Wettergren, Antonio Cosma, Anders Ståhlberg, Elinor Bexe Lindskog, Marianne Quiding Järbrink

**Affiliations:** 1 Department of Immunology and Microbiology, Institute of Biomedicine, University of Gothenburg, Sahlgrenska Academy, Gothenburg, Sweden; 2 Department of Laboratory Medicine, Sahlgrenska Center for Cancer Research, Institute of Biomedicine, University of Gothenburg, Sahlgrenska Academy, Gothenburg, Sweden; 3 Wallenberg Centre for Molecular and Translational Medicine, University of Gothenburg, Gothenburg, Sweden; 4 Department of Clinical Genetics and Genomics, Sahlgrenska University Hospital, Gothenburg, Sweden; 5 Department of Surgery, Institute of Clinical Sciences, University of Gothenburg, Sahlgrenska Academy, Gothenburg, Sweden; 6 Department of Surgery, Region Västra Götaland, Sahlgrenska University Hospital, Gothenburg, Sweden; 7 National Cytometry Platform, Luxemburg Institute of Health, Esch-sur-Alzette, Luxemburg

**Keywords:** γδ T cells, Colon cancer, Tumour immunity, TCRδ chain

## Abstract

Γδ T cell infiltration into tumours usually correlates with improved patient outcome, but both tumour-promoting and tumoricidal effects of γδ T cells have been documented. Human γδ T cells can be divided into functionally distinct subsets based on T cell receptor (TCR) Vδ usage. Still, the contribution of these different subsets to tumour immunity remains elusive. Here, we provide a detailed γδ T cell profiling in colon tumours, using mass and flow cytometry, mRNA quantification, and TCR sequencing. δ chain usage in both the macroscopically unaffected colon mucosa and tumours varied considerably between patients, with substantial fractions of Vδ1, Vδ2, and non-Vδ1 Vδ2 cells. Sequencing of the Vδ complementarity-determining region 3 showed that almost all non-Vδ1 Vδ2 cells used Vδ3 and that tumour-infiltrating γδ clonotypes were unique for every patient. Non-Vδ1Vδ2 cells from colon tumours expressed several activation markers but few NK cell receptors and exhaustion markers. In addition, mRNA analyses showed that non-Vδ1 Vδ2 cells expressed several genes for proteins with tumour-promoting functions, such as neutrophil-recruiting chemokines, Galectin 3, and transforming growth factor-beta induced. In summary, our results show a large variation in γδ T cell subsets between individual tumours, and that Vδ3 cells make up a substantial proportion of γδ T cells in colon tumours. We suggest that individual γδ T cell composition in colon tumours may contribute to the balance between favourable and adverse immune responses, and thereby also patient outcome.

**Supplementary Information:**

The online version contains supplementary material available at 10.1007/s00262-024-03758-7.

## Background

Γδ T cells are unconventional T cells expressing a semi-variable T cell receptor (TCR) composed of a limited selection of γ and δ chains, which bind to invariant MHC I-like molecules, as well as other stress-induced cell surface proteins. Cognate TCR binding leads to immediate effector functions, such as cytotoxicity and cytokine secretion. Human γδ T cells are usually characterized based on δ chain usage, where Vδ1, Vδ2, and Vδ3 are the most common. Furthermore, the preferential pairing of different δ and γ chains divide γδ T cells into additional subsets [1, 2]. Oligoclonal populations of γδ T cells are present in different tissues, such as mucosal tissues, skin, and peripheral blood [3]. In humans, Vδ2 cells dominate in the circulation, while Vδ1 cells are more common in the intestinal mucosa [1–3]. In addition to the TCR, both Vδ1 and Vδ2 cells express various NK cell receptors that react to the expression of surface molecules induced in both infected and transformed cells. Especially the expression of NKp30 and NKp46 has been shown to delineate subsets of γδ T cells with increased cytotoxic capacity towards tumour cells [4, 5]. When activated, γδ T cells also produce pro-inflammatory cytokines in addition to their cytotoxic functions [6]. In a cancer setting, the infiltration of γδ T cells has been associated with an improved clinical outcome in studies across several types of haematological malignancies and solid tumours, including colorectal cancer (CRC) [7–9]. However, in studies with a CRC focus, γδ T cells were both positively and negatively correlated to a favourable patient outcome [8, 10, 11]. Generally, anti-tumour immunity and a beneficial patient response are commonly associated with cytotoxicity and the production of Th1 type cytokines [12–14]. As conventional T cells, γδ T cells can be divided into different subsets based on cytokine production. In tumour immunity, the two best described are γδ1 and γδ17 cells, with a cytokine profile similar to Th1 and Th17 cells, respectively, and the proportions of these cells detected in different studies vary considerably [15, 16].

There is currently a lack of understanding of which γδ T cell subsets contribute to a pro- or anti-tumour immune response, and how they distribute in individual tumours. In this study, we could show that γδ T cells-infiltrating colon tumours express Vδ1, Vδ2, or Vδ3 TCRδ chains and that these subsets are distinct from circulating γδ T cells. The proportions of these cells varied considerably among tumours, as did the clonotypes detected, which were all private to a single tumour. We identified a substantial presence of Vδ3 cells in colon tumours which had reduced anti-tumour effector functions and expressed several tumour-promoting mediators.

## Material and methods

### Patient samples

This study was performed at the Sahlgrenska Academy at the University of Gothenburg together with the Sahlgrenska University Hospital. All procedures and experiments were performed in accordance with the Declaration of Helsinki and were approved by the Regional Research Ethics Committee of western Sweden (reference no 249–15). Venous blood, macroscopically unaffected colon mucosa (collected at least 10 cm away from the tumour border), and tumour tissue were collected from 45 colon cancer patients (25 males and 20 females, aged 38 to 90, median age 75) undergoing resection surgery for stage I–IV tumours. Cells from 15 of these patients were used for mass cytometry, 27 for flow cytometry analyses, and 3 for both mass and flow cytometry. See Suppl. Table S1 for additional patient and tumour characteristics. In a separate set of 10 patients, comprising 7 males and 3 females, aged 51–89 (Suppl. Table S1), we analysed the TCR repertoire in resected tumour tissues. None of the patients had undergone radio- or chemotherapy during the last 2 years. Microsatellite status was determined as previously described using the microsatellite instability (MSI) Analysis System v.1.2 (ProMega) [17]. MSI-High (MSI-H) tumours were defined as tumours with more than 1 marker showing instability, MSI-Low (MSI-L) as tumours with one marker showing instability, and microsatellite stable (MSS) tumours as tumours with no markers showing instability.

### Cell isolation and stimulation

The tissue material was collected during surgery and transported in ice-cold PBS before isolation of lymphocytes within two hours, and lamina propria lymphocytes were isolated as previously described [18]. Venous blood samples were collected in heparinized tubes during surgery, and peripheral blood mononuclear cells (PBMCs) were isolated by density-gradient centrifugation using Ficoll-Paque (GE Healthcare Bio-Sciences AB).

Enrichment of CD45^+^ cells was performed before mass cytometry using the REAlease TIL MicroBead kit (Miltenyi). Cells were kept overnight at 37 °C for functional assays and mass cytometry analysis or at 4 °C for phenotypic analysis using flow cytometry. For cytokine production analyses, cells were incubated overnight in culture medium at 37 °C before polyclonal stimulation the following morning using 50 ng/mL of phorbol 12-myristate 13-acetate (PMA) and 680 ng/mL of ionomycin calcium salt (Sigma-Aldrich) for 4 h, together with a protein transport inhibitor (BD Golgi stop, BD Biosciences).

### γδ TCR sequencing

Resected tissue samples were cut into smaller pieces and immediately frozen and stored in liquid nitrogen in advanced DMEM/F12 (Thermo Fisher) substituted with 100 U/ml of Penicillin and 100 µg /ml of Streptomycin, 10 mM HEPES, Glutamax according to the supplier’s recommendation (Gibco), and 10% dimethylsulphoxid (DMSO) until isolation of lymphocytes as previously described [19]. γδ TCR sequencing was performed on DNA extracted from the isolated lymphocytes with the QIAamp Blood Mini Kit (Qiagen), according to the manufacturer’s instructions. A total of 1.5 µg DNA was analysed using SiMSen-Seq [20], except for one patient (Patient#405) where only 1 µg DNA was available. Library quantity and size distribution were assessed on a Fragment Analyzer using HS NGS Fragment kit (Agilent Technologies). The libraries were pooled at equimolar concentration and purified with a Pippin Prepp using 2% agarose gel reagent kit (Sage Science). Final libraries were quantified with quantitative PCR and then sequenced on the MiniSeq Sequencing System using paired-end and 2 times 150 bp sequencing (Illumina). The raw sequencing data in fastq format have been deposited to the NCBI short read archive (SRA; https://www.ncbi.nlm.nih.gov/sra) with accession number PRJNA1107040.

The raw sequencing reads for γδ TCR sequencing were analysed with the MIGEC bioinformatics pipeline [21], including unique molecular identifier extraction, consensus read assembly, and annotation of the complementarity-determining region 3 (CDR3) region including annotation of V, D, and J segments, by blasting to known CDR3 sequences.

### Mass cytometry

Mass cytometry analysis was performed as previously described [22], using live cell barcoding with CD45 antibodies conjugated to different isotopes to individually label cells from blood, unaffected colon mucosa, and tumour samples [23]. For a detailed antibody list, see Suppl. Table S2.

### Flow cytometry

Single cell suspensions were stained with a live/dead exclusion dye followed by antibodies to surface antigens. For detection of cytokines and GrB, cells were fixed and permeabilised using the FoxP3 staining kit (eBioscience). For a detailed antibody list, see Suppl. Table S3. The samples were acquired on a BD LSR Fortessa. Samples with fewer than 50 cells of any of the investigated subsets (Vδ1, Vδ2, and non-Vδ1Vδ2 cells) were not included in the phenotypic or functional analyses.

### mRNA quantification

Live Vδ1, Vδ2, and non-Vδ1Vδ2 cells from 4 colon tumours were sorted using a BD FACS-Aria Fusion. Multiplex mRNA quantification was performed using the nCounter Analysis system together with the nCounter human Immunology v2 panel (Nanostring) at KIGene (Karolinska Institutet, Stockholm). Nanostring data were normalized to adjust for platform-associated and sample input variations and thresholds were set according to Nanostring guidelines (0, 3–3 for the positive control normalization and 0, 1–10 for the housekeeping gene normalization). The Vδ1 cells from one patient were subsequently excluded from analysis due to low RNA quality. The normalized data have been deposited to Gene Expression Omnibus (GEO; https://www.ncbi.nlm.nih.gov/geo/) with accession number GSE266504.

### Immunofluorescence

Four-μm cuts from formalin-fixed paraffin-embedded tissue blocks of unaffected mucosa and colorectal tumours were mounted on Superfrost Plus microscope slides. Sections were deparaffinized and rehydrated, and antigens were unmasked with pH9 Tris–EDTA buffer. Tissue was stained with CD3 (A0452, Agilent; Opal 570), CD8α (SP16, Thermo Fisher Scientific; Opal 620), TCRδ (H41, Santa Cruz Biotechnologies; Opal 690), pan-cytokeratin (KRT/1877R, Abcam; Opal 520), respectively, using the Opal Polaris 7-Color Manual IHC Kit (Akoya Biosciences). Subsequently, nuclei were stained with spectral DAPI (Akoya Biosciences) and slides were mounted with ProLong Glass Antifade Mounting media (Thermo Fisher Scientific). Tissue sections were scanned with the Metafer Slide Scanning Platform (Axio Imager.Z2 Microscope and 20x/0.8/air objective, Zeiss) equipped with a SpectraSplit filter system (Kromnigon). Images were analysed with Strataquest (TissueGnostics).

### Data processing and statistical analysis

Mass cytometry data were analysed using OMIQ version 10. All clusters that contributed with less than 1% of all γδ T cells were excluded from the analysis. Data from the multiplex mRNA quantification were analysed using the Nsolver software (version 4). Flow cytometry data were analysed using FlowJo version 10 and OMIQ version 10. Gini-Simpson diversity index was calculated using the Diverse package in R (version 0.1.5). Statistical analyses of paired data were performed using two-tailed Wilcoxon matched-pairs signed rank test and of unpaired data using two-tailed Mann–Whitney test. When comparing three groups of matched data, the Friedman test followed by Dunn’s post-test was used to achieve multiplicity adjusted P values. Statistical tests were performed using GraphPad PRISM version 9. *P*-values < 0.05 were considered statistically significant.

## Results

### γδ T cells in colon tumours

**Fig. 1:**
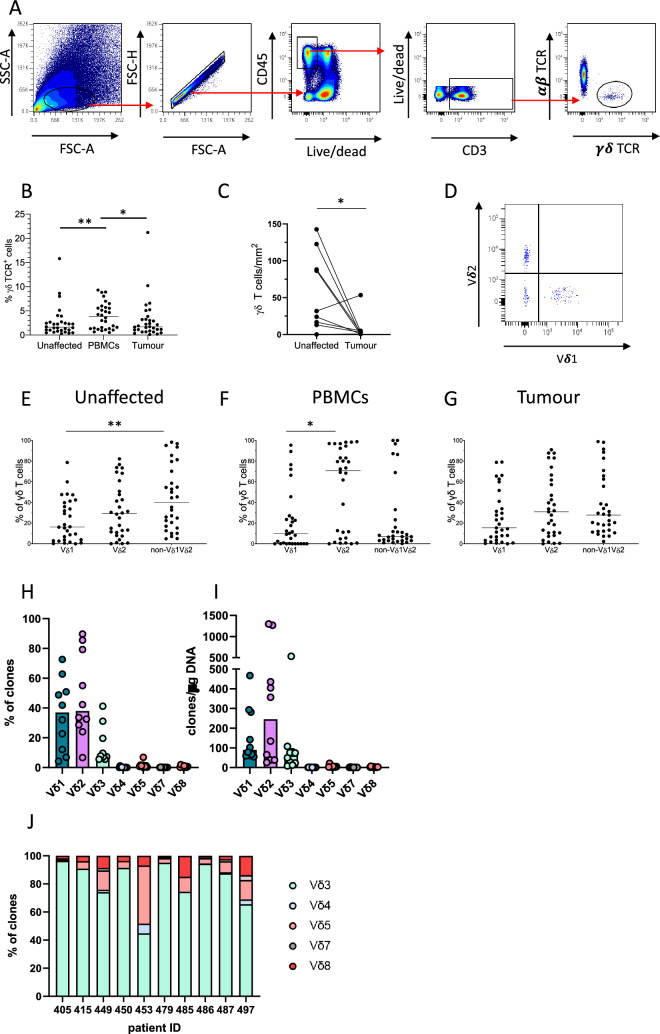
Identification of tumour-infiltrating γδ T cells. Single cell suspensions were isolated from tumours, corresponding unaffected colon mucosa, and blood, and the frequencies of γδ T cells among the CD3^+^ T cells were analysed using flow cytometry, immunofluorescence, and CDR3 sequencing. **A** Gating strategy from a representative tumour sample. **B** Frequencies of γδ T cells among all CD3^+^ lymphocytes determined by flow cytometry. **C** Density of γδ T cells determined by fluorescence microscopy in sections from formalin-fixed tumours and corresponding unaffected colon mucosa. **D** Flow cytometry staining of Vδ1 and Vδ2 in a representative tumour sample of γδ T cells gated as in (a). **E**–**G** Usage of the Vδ1 and Vδ2 chains by γδ T cells was determined by flow cytometry in cell suspensions from unaffected colon mucosa (**E**), blood (**F**), and tumour (**G**). **H** Vδ chain usage was determined by CDR3 sequencing in γδ T cells isolated from colon tumours and the percentage of clones using the respective Vδ segments or (**I**) the number of clones using each Vδ segment per μg of DNA is shown for each patient. In (**I**) values less than 1 were set at 1 to improve visualization. **J** Distribution of non-Vδ1Vδ2 clones in the individual tumours. Symbols represent individual values and lines the median. In **(C)**, symbols are connected to show corresponding values from the same patients. Data in (**B**), (**E**), (**F**), and (**G**) were analysed using two-tailed Friedman test followed Dunn’s post-test and in (**C**) using two-tailed Wilcoxon test. *p < 0.05 and ** < 0.01. n = 10 for immunofluorescence and CDR3 sequencing, and n = 30 for flow cytometry analyses

To investigate the subsets of γδ T cells present in colon tumours, we used fresh samples recovered from patients undergoing resection surgery. Using flow cytometry, γδ T cells were identified as CD3^+^ cells stained by a pan-γδ-TCR antibody but not by a pan-αβ-TCR antibody (Fig. 1A). γδ T cell frequencies were significantly lower in both the tumours and the macroscopically unaffected colon compared to in the blood (Fig. 1B). Using fluorescence microscopy, we also analysed the numbers of γδ T cells present in the tumours and unaffected colon mucosa. Here, the numbers of γδ T cells were also significantly reduced (*p* < 0.05) in the tumour compared to the unaffected colon mucosa from the same individuals (Fig. 1C, Suppl. Fig. S1). These analyses also showed that γδ T cells in the tumours were primarily positioned in the stroma rather than in the tumour epithelium.

To further define the TCRs of tumour-infiltrating γδ T cells, we analysed the Vδ chain usage (Fig. 1D). The dominating subset in the circulation was Vδ2 cells. In the tissue, there were also considerable numbers of Vδ2 cells in both unaffected mucosa and tumours, while Vδ1 cells were less numerous in most patients. We could also document a substantial proportion of γδ T cells that did neither express Vδ1 nor Vδ2. These non-Vδ1Vδ2 cells were present in both tumour and unaffected mucosa from all patients (Fig. 1E–G). Quantitative Vδ CDR3 sequencing analyses clearly showed that the large majority of non-Vδ1 Vδ2 cells in the tumours used Vδ3. Only one out of ten patients displayed a sizeable Vδ5 population alongside the Vδ3 cells (Fig. 1H, I, J).

**Fig. 2:**
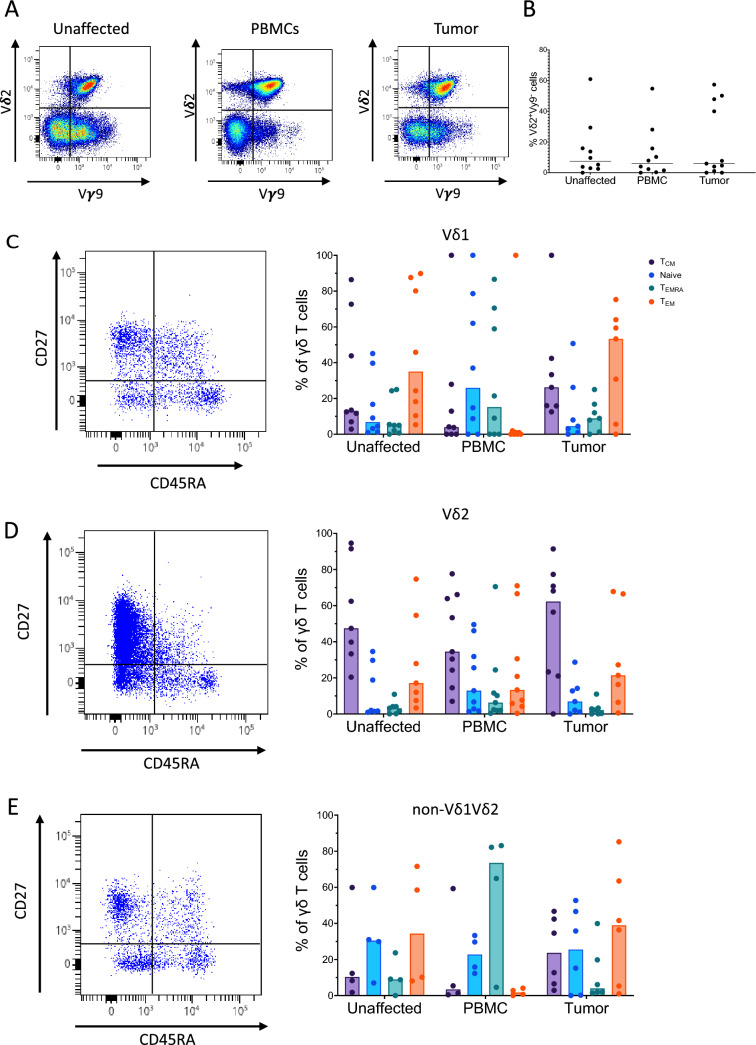
Phenotype of tumour-infiltrating γδ T cells. Single cell suspensions were isolated from tumours, corresponding unaffected colon mucosa, and blood, and analysed using flow cytometry. **A** Vδ2 and Vγ9 staining in an unaffected tissue, blood, and tumour from a representative patient. **B** Frequencies of Vγ9^−^ cells among all Vδ2 cells from the different tissues. **C–E** The frequencies of central memory, naïve, terminally differentiated effector memory, and effector memory cells among the Vδ1 (**C**), Vδ2 (**D**), and non-Vδ1Vδ2 (**E**) γδ T cells are shown alongside dot plots of Vδ1, Vδ2, and non-Vδ1Vδ2 cells from tumour tissue. Symbols represent individual values and lines and bars the median. n = 4–9

Vδ2 cells are divided into two main types based on their usage of the Vγ9 chain. The classical, innate-like Vγ9^+^Vδ2^+^ cells are the most common, while the rarer Vγ9^−^Vδ2^+^ cells have been described as a more adaptive-like cell type with a more diverse TCR [24]. γδ T cells expressing the Vγ9 chain were common in all tissues, and Vγ9 was most commonly paired with Vδ2 (Fig. 2A). However, we also found fractions of both Vδ1 and non-Vδ1Vδ2 cells in all tissues that expressed the Vγ9 chain (Suppl. Fig. S2). Vγ9^−^Vδ2^+^ cells were present to some extent in tissue samples and blood from most patients (Fig. 2B). We also detected low to moderate expression of CD8 in all subsets of γδ T cells investigated in all the tissues examined (Suppl Fig. S3).

Different naïve and memory populations of γδ T cells can be distinguished based on the expression of CD45RA and CD27, defining naïve (CD45RA^+^CD27^+^), central memory (T_CM_, CD45RA^−^CD27^+^), effector memory (T_EM_, CD45RA^−^CD27^−^), and terminally differentiated effector memory (T_EMRA_, CD45RA^+^CD27^−^) cells [25]. This classification was originally devised for conventional αβ T cells and may not be directly applicable to γδ T cells, but we have used it here for convenience. The Vδ1 cells in the tumours and unaffected mucosa were usually dominated by T_EM_ cells, while circulating Vδ1 cells were dominated by naïve and T_EMRA_ cells (Fig. 2C). Vδ2 cells were similar to each other in all the examined locations and dominated by cells with a T_CM_ phenotype (Fig. 2D). In the non-Vδ1Vδ2 cells in the colon mucosa and the tumours, the naïve cells were more prominent than in Vδ1 and Vδ2, and there was also a strong component of T_EM_ cells in the non-Vδ1Vδ2 subset. In addition, the circulating non-Vδ1Vδ2 cells were dominated by T_EMRA_ cells (Fig. 2E).

Taken together, these results show that γδ T cells do not infiltrate colon tumours to the same extent as the surrounding unaffected colon mucosa, but that there is a prominent subset of non-Vδ1Vδ2 cells primarily made up of Vδ3 cells in the tumours.

### Infiltration of non-Vδ1Vδ2 cells in relation to clinicopathologic features

**Fig. 3:**
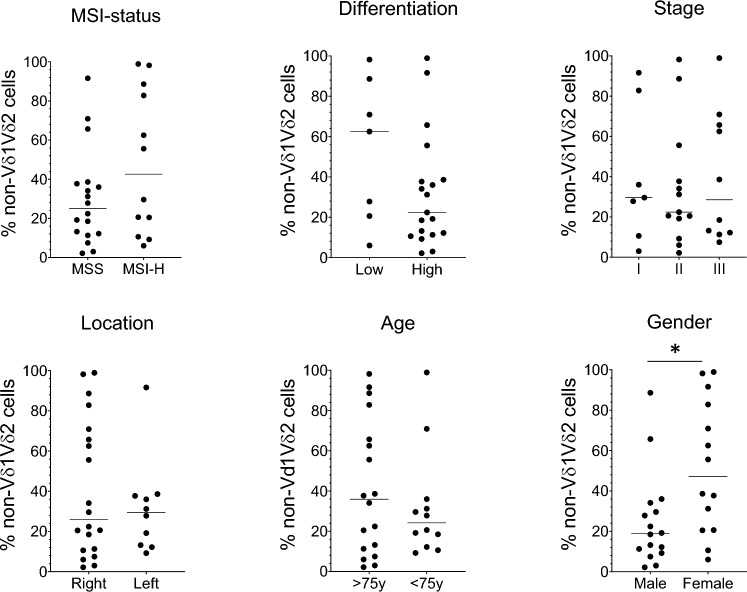
Tumour-infiltrating non-Vδ1Vδ2 cells and tumour characteristics. Single cell suspensions were isolated from tumours, and the frequencies of non-Vδ1Vδ2 cells among the γδ T cells were determined by flow cytometry and related to MSI status, tumour differentiation, stage, and location, and patient age and gender. Symbols represent individual values and lines the median. Data were analysed using two-tailed Wilcoxon test. *p < 0.05. n = 30

As the size of the non-Vδ1Vδ2 subset varied considerably between patients, we were interested to relate their presence to clinicopathologic features. However, in this relatively small material there was no correlation between non-Vδ1Vδ2 cell proportions and MSS/MSI status, tumour differentiation, stage, location (right vs left sided), or patient age. Only when comparing men and women could we find a significantly higher proportion of non-Vδ1Vδ2 cells in the tumours from female patients (Fig. 3).

### Clonality of tumour-infiltrating γδ T cells

**Fig. 4:**
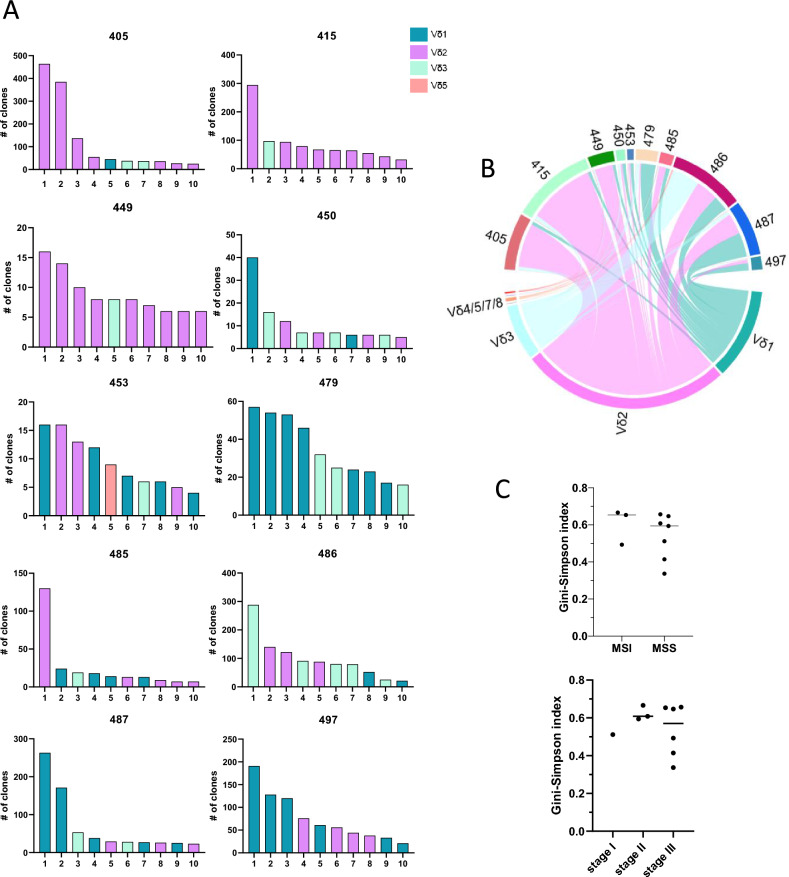
Vδ chain usage in tumour-infiltrating γδ T cells. Single cell suspensions were isolated from frozen tumour specimens and the CDR3 region analysed with ultra-sensitive sequencing using unique molecular identifiers. **A** The number of clones in the ten most frequent clonotypes from each patient. Colour coding shows the Vδ usage in the respective clonotypes. **B** Chord diagram showing the distribution of clones using the different Vδ chains in individual patients. **C** Gini-Simpson index of diversity was calculated for each tumour and plotted as a function of microsatellite status and tumour stage. Symbols represent individual values and the line the median. n = 10

To determine clonality, and the potential of public, shared γδ clonotypes between patients, the δ chain CDR3 sequence was analysed. δ chain sequencing of 10 colon tumours resulted in a total of 9,403 productive recombinations, representing individual cells, where the CDR3 sequence was reliably determined, ranging from 175 to 2,167 recombinations from individual tumours. These recombinations were distributed between 2,092 clonotypes, containing between 1 and 464 recombinations per clonotype. The distribution of clonotypes differed markedly between individual tumours (Fig. 4A). Of note, the dominating clonotypes were either Vδ1, Vδ2, or Vδ3 in different tumours. However, in all but one of the patients, Vδ3 cells made up one to five of the ten dominating clonotypes (Fig. 4A, B). Unfortunately, we did not have access to unaffected tissue and blood from these individuals and could thus not investigate to which extent these clones were present in healthy tissues. The difference in γδ T cells between individual tumours was also reflected in the Gini-Simpson diversity index, which varied between 0.49 and 0.77 in the different tumours. There was no difference in diversity between cells from MSI-L/H and MSS tumours or between different stage tumours (Fig. 4C). Furthermore, there was no overlap between the clonotypes found in any patients, further emphasizing the large interindividual variation in γδ T cell composition between patients.

### Mass cytometry and mRNA quantification reveal diverse clusters of tumour-infiltrating γδ T cells

**Fig. 5:**
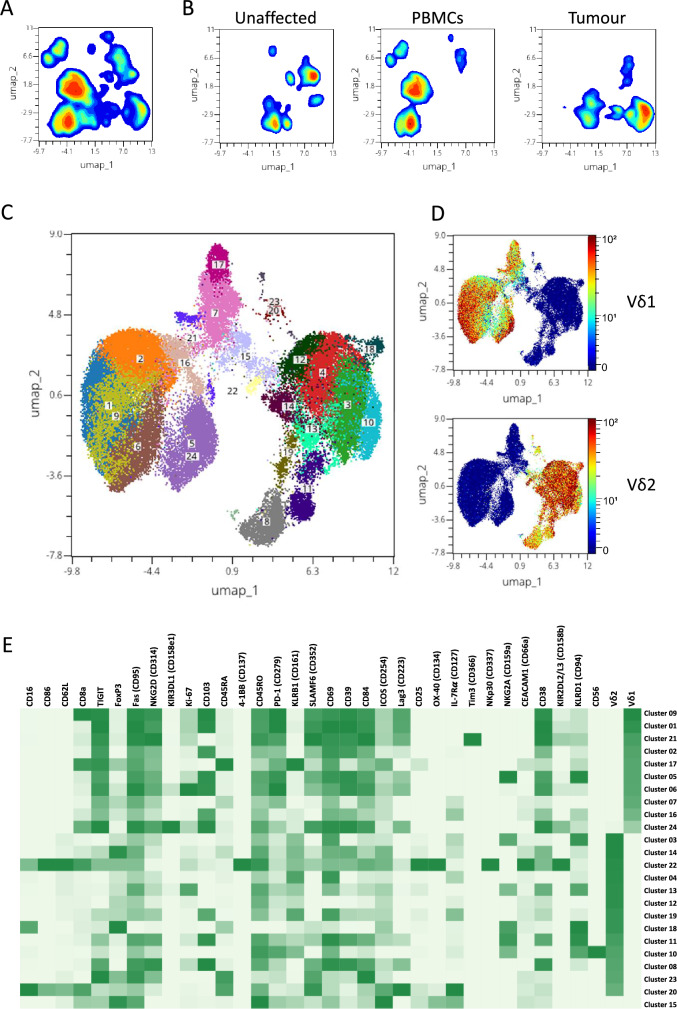
Clustering analysis of tumour-infiltrating γδ T cells. Single cell suspensions were isolated from tumours, corresponding unaffected colon mucosa, and blood, and analysed using mass cytometry. **A** γδ T cells were first analysed using the UMAP dimensional reduction algorithm in concatenated data combined from blood, tumour, and unaffected tissue. **B** Data from **A** are shown individually for unaffected tissue, PBMCs, and tumours. **C** Tumour-infiltrating γδ T cells were analysed separately using the UMAP dimensional reduction algorithm together with the phenograph clustering algorithm. The markers indicated in (**E)** were all used to generate the clustering algorithms. **D** Expression of Vδ1 and Vδ2 overlaid on the clustered tumour-infiltrating γδ T cells. The colour scale represents staining intensity, and the scale is based on the minimum to the maximum signal in each specific marker. **E** Heatmap of marker expression in the clusters identified in tumour-infiltrating γδ T cells using the UMAP dimensional reduction and phenograph clustering algorithms. The colour scale shows the median signal intensity of the respective marker in each cluster, and the scales were generated individually for each marker and based on the minimum to the maximum signal in each specific marker. n = 18

To gain additional understanding of the different subsets of γδ T cells beyond δ chain usage, we employed a panel of antibodies focused on cytotoxicity and exhaustion markers and analysed *ex-vivo* isolated T cells using mass cytometry. γδ T cells were manually gated as live CD45^+^CD3^+^CD4^−^TCR γδ^+^ cells, and unsupervised dimensional reduction of the aggregated data from all patients was performed using the UMAP algorithm, followed by clustering using the phenograph algorithm. Initially, we clustered 59,110 γδ T cells from tumours, 38,275 from unaffected colon mucosa, and 204,561 from PBMC collected from 18 patients (Fig. 5A). From these analyses, it was clear that γδ T cells from blood and the colon tissue formed distinct clusters with low or no overlap (Fig. 5B). As the tumour-infiltrating lymphocytes presumably are the most relevant for anti-tumour immunity, we subsequently focused on their phenotype and effector functions. We thus performed unsupervised analysis of 59,110 tumour-infiltrating γδ T cells (Fig. 5C). Based on the expression of the Vδ1 or Vδ2 chain, 3 distinct groups containing Vδ1, Vδ2, and non-Vδ1Vδ2 cells were observed (Fig. 5D) In the tumours, we could identify 10 clusters of Vδ1 cells, 13 clusters of Vδ2 cells, and a single cluster of non-Vδ1Vδ2 cells (cluster 15). Expression of individual markers across the UMAP projection is shown in Suppl. Fig. S4. The contribution of cells from individual tumours to a certain cluster differed. Most clusters were made up of cells from all the tumours, while some clusters (e.g. cluster 18 and 20) consisted mainly of cells from a single tumour (Suppl. Fig. S5). While expression of several markers could be found in most clusters present in the tumours, other markers varied substantially in expression. For instance, the Vδ1 cells generally had a higher expression of CD103, CD38, and the exhaustion markers TIGIT, PD-1 (CD279), and CD39 compared to the other subsets. In contrast, the Vδ2 clusters were much more diverse with expression of several markers unique to only one or two clusters (Fig. 5E). The single non-Vδ1Vδ2 cluster present in the tumours had a high expression of CD45RO and Fas (CD95), and also a higher expression than most other clusters of several proteins expressed in activated cells, such as ICOS (CD254), OX-40 (CD134), CD25, and FoxP3 (Fig. 5E).

**Fig. 6:**
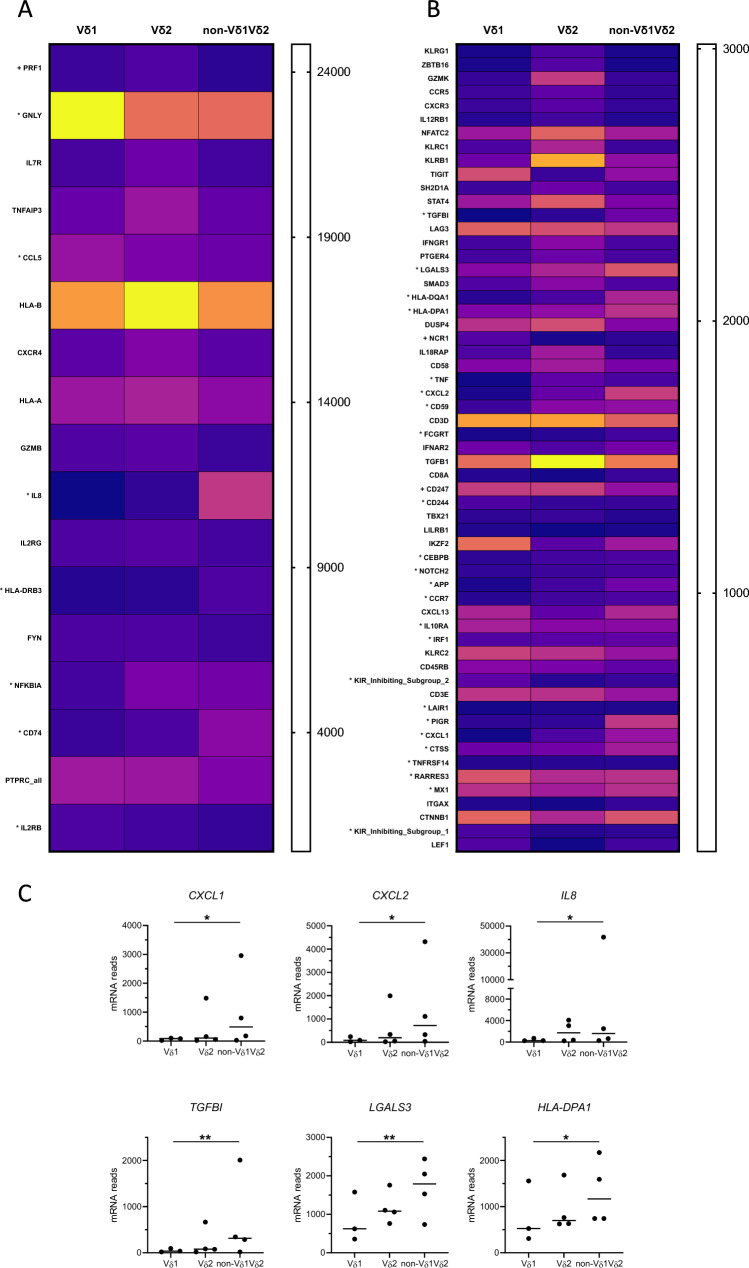
mRNA expression in tumour-infiltrating γδ T cell subsets. Single cell suspensions were isolated from tumours and Vδ1, Vδ2, and non-Vδ1Vδ2 cells were sorted by flow cytometry and analysed using multiplex mRNA quantification. To facilitate interpretation of the data, the heatmap of differentially expressed genes has been divided into mRNAs with a high (**A**) and low (**B**) expression, respectively. Genes are presented in order of the highest to the lowest significance values of the difference between non-Vδ1Vδ2 cells and the other subsets within the two panels. The intensity scales indicate the normalized counts of mRNAs per cell. Genes marked with * indicate significant differences between non-Vδ1Vδ2 and Vδ1 cells, while no marking indicates significant differences between non-Vδ1Vδ2 and Vδ2 cells. **C** mRNA counts in cell subsets from individual tumours are shown for *CXCL1*, *CXCL2*, *IL8*, *TGFBI*, *LGALS3*, and *HLA-DPA1*. Data were analysed using two-tailed Friedman test without adjustment for multiple comparisons. *p < 0.05, **p < 0.01. n = 4

In a separate set of four patients, the tumour-infiltrating Vδ1, Vδ2, and non-Vδ1Vδ2 cells were sorted by flow cytometry immediately after isolation, and mRNA quantified. In total, we identified 76 genes that were differentially expressed between non-Vδ1Vδ2 and Vδ1 or Vδ2 cells (Suppl. Table S4). Vδ1 cells presented a signature consistent with cytotoxic effector functions, with a high expression of *GNLY* (Granulysin), *PRF1* (Perforin), *CD244* (2B4), and *NCR1* (NKp46) (Fig. 6A, B). A cytotoxic effector signature could also be observed when comparing the Vδ2 and the non-Vδ1Vδ2 cells. However, in addition to *PRF1*, the Vδ2 transcriptome was dominated by expression of *GRZK* and *GRZB* (granzymes K and B), the killer cell lectin-like receptors *KLRG1*, *KLRC1* (NKG2A), and *KLRB1* (CD161; Fig. 6A, B). Interestingly, the non-Vδ1Vδ2 cells had a higher expression of genes associated with inflammatory and tumour-promoting responses, such as *CXCL1* (GRO-α), *CXCL2* (GRO-β), *IL8* (IL-8), *TGFBI* (transforming growth factor-beta induced, TGFBI), and *LGALS3* (Galectin-3), and several genes associated with antigen presentation (*HLA-DPA1, HLA-DQA1, HLA-DRB3,* and *CD74*), when compared to the other subsets (Fig. 6C).

Taken together, these analyses show that Vδ1 and Vδ2 cells express markers that are associated with cytotoxic effector functions. In contrast, the non-Vδ1Vδ2 cells appear to have a more tumour-promoting function, as they express less NK cell receptors and cytotoxic effector molecules, but instead markers associated with an innate inflammatory immune response and direct tumour-promoting functions.

### Cytokine production in tumour-infiltrating γδ T cells

**Fig. 7:**
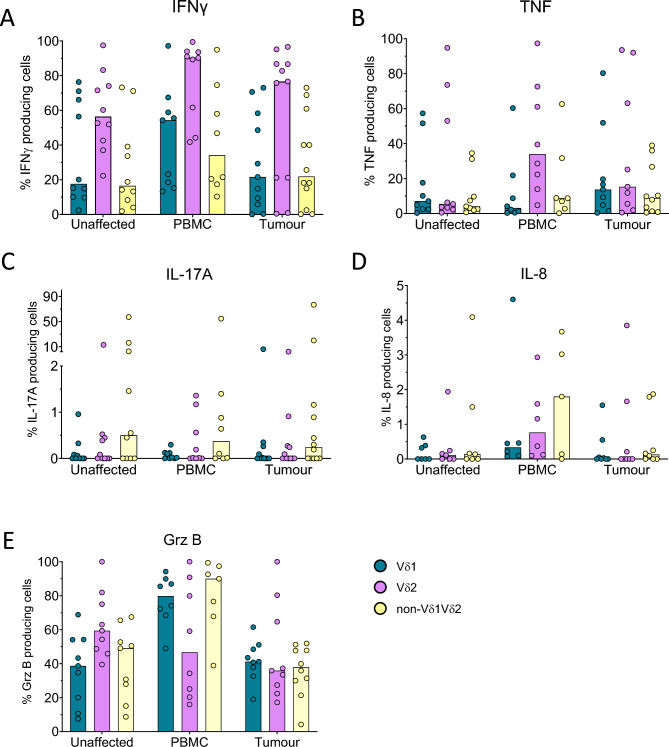
Cytokines and effector proteins in tumour-infiltrating γδ T cells. Single cell suspensions were isolated from tumours, corresponding unaffected colon mucosa, and blood, and stimulated with PMA and Ionomycin. Vδ1, Vδ2, and non-Vδ1Vδ2 cells were analysed for the expression of IFN-γ (**A**), TNF (**B**), IL-17A (**C**), IL-8 (**D**), and Granzyme B (**E**) by flow cytometry. Symbols represent individual values and the bars the median. n = 5–12

To better understand the functional capacity of the non-Vδ1Vδ2 cell subset in colon cancer patients, we analysed the production of Th1 and Th17 associated cytokines and GrB following polyclonal stimulation. These experiments revealed that IFN-γ was highly expressed especially in Vδ2 cells from all tissues. In contrast, the non-Vδ1Vδ2 cells from the unaffected colon mucosa and the tumours only contained moderate frequencies of IFN-γ-producing cells (Fig. 7A). TNF production was considerably lower than that of IFN-γ and was also lower in the non-Vδ1Vδ2 subset compared to the Vδ2 subset in the cells present in both the tissue and the circulation (Fig. 7B). In contrast, IL-17A expression was only seen in non-Vδ1Vδ2 cells from some individuals, but virtually not in any of the other subsets of γδ T cells (Fig. 7C). This was similar to IL-8 expression, which was only detected in some patients and primarily in circulating non-Vδ1Vδ2 cells (Fig. 7D). GrB, on the other hand, was expressed at relatively high levels in cells from all tissues from all patients. Furthermore, there were no substantial differences in GrB production between the γδ T cell subsets from the tumours using different TCRs (Fig. 7E). Representative flow cytometry plots from one patient can be found in Suppl. Fig. S6–S8. The median fluorescence intensity of the cells staining positive for the respective cytokines was generally similar between the γδ T cells subsets, except for GrB staining intensity which was especially high in Vδ1 and non-Vδ1 Vδ2 cells from the circulation (Suppl. Fig. S9). In general, GrB production from γδ T cells was higher than in conventional αβ T cells, while TNF and IL-17 production was lower and IFN-γ and IL-8 production was similar to that in αβ T cells (Suppl. Fig. S10).

## Discussion

Recent studies in CRC show the presence of different subsets of tumour-infiltrating γδ T cells with specific functions, which range from tumour-promoting to tumoricidal effects [11, 26]. This is likely context dependent and is yet to be fully understood. In this study, we used several strategies to delineate different subpopulations of tumour-infiltrating γδ T cells in colon cancer patients. We show that γδ T cell infiltration into tumours was reduced in most patients, compared to the surrounding unaffected colon mucosa, and that the tumour-infiltrating γδ T cells vary considerably between patients with regard to Vδ chain usage, phenotype, and functional properties.

Most research on human γδ T cells has focused on Vδ1 and Vδ2 cells, mainly due to the limited availability of antibodies to the other TCRs. However, in human tissues there is a considerable proportion of γδ T cells using other Vδ chains, both in tumours and the corresponding healthy tissue [5, 11, 26–28]. Here, we could document a similar accumulation of non-Vδ1Vδ2 cells in human colon tissues. In the tumours, TCR sequencing showed that these cells expressed Vδ3 to a very large extent and also contributed to the most expanded clonotypes in most of the patients. The Vδ3 cells in the tumours were often oligoclonal with one or a few dominating clones, and they may recognize tumour neoantigens or stress signals in the tumour cells, such as Annexin A2 [29]. The cognate ligands for Vδ3 cells also include the monomorphic MHC I-like molecules CD1d and MR1 [30, 31]. These molecules are also increased on the cell surface following endoplasmatic reticulum (ER) stress and inflammatory signals [32, 33], and reactivity against such antigens may also explain some of the clonal expansion of Vδ3 cells in the tumours.

We have used CD27 and CD45RA as markers of different memory populations, even though this nomenclature was originally devised for αβ T cells. Vδ1 and non-Vδ1 Vδ2 cells from colon, both unaffected and tumour tissue, harboured a large proportion of T_EM_-like cells which were not present among the circulating cells. This is similar to tissue-infiltrating γδ T cells in liver tissue and non-small cell lung cancer, where similar T_EM_-like Vδ1 cells have been documented [34, 35]. In the Vδ1 and non-Vδ1 Vδ2 cells, the distribution between memory subsets was conserved in colon mucosa and tumours, but different in blood, while Vδ2 cells were similar with regard to memory subsets in blood and tissues. Therefore, we cannot rule out the possibility that a substantial proportion of the Vδ2 cells detected in the colon tissues may in fact originate from the microvasculature, while Vδ1 and non-Vδ1 Vδ2 cells might more likely represent tissue-resident cells, as previously documented in lung and ovarian cancer [35, 36].

Both Vδ1 and Vδ2 cells have been attributed potent anti-tumour effects, while the effect of other γδ T cells in the tumour microenvironment is more elusive [37]. Vδ1 cells possess potent cytotoxic activity towards cancer cells in vitro and a high expression of cytotoxic effector proteins, such as Granzyme B [5, 38]. Previous detailed transcriptional analyses of tumour-infiltrating γδ T cells revealed distinct clusters based on the transcriptional profiles of Vδ1 and Vδ2 cells that exhibited similar expression of cytotoxic markers as the clusters of CD8^+^ T cells and NK cells [28]. In our study, we identified several clusters of both Vδ1 and Vδ2 cells with both overlapping and unique features. A distinct feature of Vδ1 and Vδ2 cells from both cell surface staining and mRNA quantification was a strong cytotoxic profile comprising both NK cell receptors and cytotoxic effector molecules. Still, cytotoxic molecules and NK cell receptors were partly differentially expressed, as previously described [28, 39]. Using a mass cytometry panel, all non-Vδ1Vδ2 γδ T cells formed a single and relatively small cluster. This is somewhat different to the flow cytometry results and may be explained by the less distinct signals in mass compared to flow cytometry. The non-Vδ1Vδ2 cells were characterized by a low surface expression of NK cell receptors and also appeared to be more activated, while they showed little sign of exhaustion. Non-Vδ1Vδ2 cells also had higher mRNA expression of neutrophil-recruiting chemokines, a tumour-promoting factor [7]. Furthermore, one of the genes we identified as more highly expressed by non-Vδ1Vδ2 cells was *TGFBI*. TGFBI has been implicated in tumour progression, and elevated levels have been associated with a poor clinical outcome, as it promotes angiogenesis and tumour cell migration, not least in CRC [40], and also reduces T cell activation [41, 42]. The expression of Galectin-3 in non-Vδ1Vδ2 cells is also interesting, as recent studies link Galectin-3 production to a poor patient outcome in CRC, increased metastatic potential, and to a γδ17 phenotype, both in healthy tissues and tumours [43, 44]. A cluster of expanded γδ T cells with high expression of Galectin-3 and other IL-17 associated genes was also recently found in human CRC tumours using single-cell RNA sequencing [26].

Functional analyses of the non-Vδ1Vδ2 cells revealed that they had a much lower expression of IFN-γ and TNF than Vδ2 cells, suggesting a lower capacity to support anti-tumour immunity. Additionally, non-Vδ1Vδ2 cells were the main source of IL-17A among γδ T cells, even though the production was limited compared to other cytokines. This is consistent with a study by Harman et al. [11], who also found IL-17-producing γδ T cells among Vδ3 cells. The restriction of IL-17 to non-Vδ1Vδ2 cells is also in line with murine studies, where distinct γδ T cell subsets provide IL-17 in the tumour microenvironment [26, 45]. Generally, intratumoural IL-17 production has been associated with a poor prognosis [8, 12], but the source of intratumoural IL-17 is not yet fully resolved [27]. Based on our current results and previous literature, it is likely that a major part of IL-17 produced in the tumour microenvironment is provided by CD4^+^ Th17 cells, rather than γδ17 cells [35, 46]. Likewise, TNF production from γδ T cells may not be crucial for the overall cytokine balance in colon tumours, while γδ T cells produce IFN-γ to an extent comparable to or higher than conventional αβ T cells.

This is a single-centre study with a well-defined patient cohort. However, one limitation of the study is the relatively small number of patients included, and the varying number of patients used for different analyses. The latter was caused by several samples containing quite few γδ T cells, and we thus had to prioritize between assays. With a larger cohort, we might have been able to detect correlations between γδ T cell subsets or functions and patient outcome.

In summary, this study demonstrates a large variation in γδ T cell composition between individual tumours with regard to phenotypic markers, functional potential, and TCR usage. Recent studies clearly demonstrate both antitumour and tumour-promoting functions of tumour-infiltrating γδ T cell subsets, which were distinguished based on TCR usage [11, 26]. Our results show substantial infiltration of non-Vδ1Vδ2 cells, primarily using Vδ3, in colon tumours and based on their low expression of cytotoxic molecules combined with higher expression of some tumour-promoting mediators, we suggest that they contribute mainly to a tumour-promoting immune response.

### Supplementary Information

Below is the link to the electronic supplementary material.

Supplementary file1 (PDF 2770 KB)

## Data Availability

TCR sequencing raw data in fastq format have been deposited to the NCBI short read archive (SRA; https://www.ncbi.nlm.nih.gov/sra) with accession number PRJNA1107040. Normalized Nanostring data have been deposited to Gene Expression Omnibus (GEO; https://www.ncbi.nlm.nih.gov/geo/) with accession number GSE266504.

## Abbreviations

CDR3: Complementarity-determining region 3
CRC: Colorectal cancer
MSI: Microsatellite instable
MSS: Microsatellite stable
PBMC: Peripheral blood mononuclear cells
PMA: Phorbol 12-myristate 13-acetate
TCR: T cell receptor
TGFBI: Transforming growth factor-beta induced
