# Supplementary material for: γδ T cells in human colon adenocarcinomas comprise mainly Vδ1, Vδ2, and Vδ3 cells with distinct phenotype and function
Source: Cancer Immunol Immunother. 2024 Jul 2;73(9):174. doi: 10.1007/s00262-024-03758-7 (PMC11219682; doi:10.1007/s00262-024-03758-7)
Supplement: Supplementary file 1 — Supplementary file1 (PDF 2770 KB) [file 262_2024_3758_MOESM1_ESM.pdf]

Supplementary table S1. Patient and tumour characteristics

	Flow and mass cytometry		TCR sequencing	
**Age**, median *(range)*	75	(38-90)	74	(51-89)
**Gender**, *n* (%)				
Male	25	(56)	7	(70)
Female	20	(44)	3	(30)
**Primary tumour location**, *n* (%)				
Right colon	28	(62)	6	(60)
Left colon	17	(38)	4	(40)
**Tumour stage**, *n* (%)				
I	9	(20)	1	(10)
II	12	(27)	3	(30)
III	22	(49)	6	(60)
IV	2	(4)	-	
**Tumour differentiation**, *n* (%)				
High	26	(58)	3	(30)
Medium	1	(2)	-	
Low	15	(33)	7	(70)
Mucinous	1	(2)	-	
Medullary	2	(4)	-	
**Microsatellite stability**, n (%)				
MSS	30	(67)	7	(70)
MSI-L	-		1	(10)
MSI-H	15	(33)	2	(20)

Supplementary table S2. CyTOF antibody panel.

Isotope	Marker	Clone	Additional info
Pt194	CD45	H130	Normal
Pt198	CD45	H130	Tumour
89Y	CD45	H130	Blood
143Nd	CD352 (NTB-A)	NT-7	
144Nd	CD69	FN50	
148Nd	CD278/ICOS	C398.4A	
149Sm	CD127 (IL-7Ra)	A019D5	
150Nd	CD223/LAG-3	11C3C65	
151Eu	CD103	Ber-ACT8	
152Sm	TCRγδ	11F2	
153Eu	TCR Vα7.2	3C10	
154Sm	TIM-3	F38-2E2	
155Gd	CD45RA	HI100	
158Gd	CD137/4-1BB	4B4-1	
159Tb	CD337 (NKp30)	Z25	
160Gd	CD28	CD28.2	
164Dy	CD95/Fas	DX2	
165Ho	CD45RO	UCHL1	
166Er	CD314 (NKG2D)	ON72	
167Er	CD158e1 (KIR3DL1, NKB1)	DX9	
169Tm	CD159a (NKG2A)	Z199	
171Yb	CD66a/CEACAM1	CD66a-B1.1	
172Yb	CD38	HIT2	
173Yb	CD158b (KIR2DL2/L3, NKAT2)	DX27	
174Yb	CD94	HP-3D9	
175Lu	CD279 (PD-1)	EH12.2H7	
176Yb	CD56 (NCAM)	NCAM16.2	
209Bi	CD16	3G8	
106 Cd	CD86	IT2.2	
110 Cd	Vδ2	B6	
111 Cd	CD62L	DREG-56	
113 Cd	CD3	SK7	
116 Cd	CD8a	RPA-T8	
161Dy	TIGIT	MBSA43	
145Nd	CD39	A1	
146Nd	CD84	CD84.1.21	
147Sm	CD134 (OX40)	ACT35	
141Pr	CD25 (IL-2R)	2A3	
142Nd	CD161	HP-3G10	
	Vδ1	REA173	PE-labelled
	CD4	2ST8.5H7	FITC-labelled
156Gd	anti PE		
Cd112	anti FITC		
162Dy	FoxP3	259D/C7	
168Er	Ki-67	B56	

Supplementary table S3. Antibodies used in flow cytometry.

Marker	Fluorochrome	clone	supplier
CD3	APC-H7	SK7	BD
CD8a	BUV395	RPA-T8	BD
CD27	BB700	M-T271	BD
CD45	AF700	H130	BD
CD45RA	BV480	HI100	BD
GrB	AF700	GB11	BD
IFN-γ	PE-CF594	B27	BD
IL-8	PE	E8N1	BD
IL-17A	BV785	BL168	BD
TCR αβ	PE-Dazzle594	IP26	Biolegend
TCR γδ	BV421	11F2	BD
TNF	BV650	MAb11	BD
Vy9	PE-Cy7	B3	BD
Vδ1	PE	REA173	Miltenyi
Vδ2	APC	123R3	Miltenyi

Supplementary Table S4. Summary of mRNA expression in Vδ1, Vδ2, and non-Vδ1Vδ2 cells

gene	directionality	Mean fold difference	p-value*	gene	directionality	Mean fold difference	p-value
**PRF1**	Vδ2>non-Vδ1Vδ2<br>Vδ1>non-Vδ1Vδ2	3.03<br>1.71	0,00156<br>0,0297	HLA-DPA1	Vδ1<non-Vδ1Vδ2	0.45	0.0132
**GNLY**	Vδ1>non-Vδ1Vδ2	2.50	0.0054	DUSP4	Vδ2>non-Vδ1Vδ2	1.88	0.0140
**IL7R**	Vδ2>non-Vδ1Vδ2	1.76	0.00637	NCR1	Vδ1>non-Vδ1Vδ2<br>Vδ2<non-Vδ1Vδ2	3.89<br>0.35	0.0177<br>0.0480
**TNFAIP3**	Vδ2>non-Vδ1Vδ2	1.61	0.00744	IL18RAP	Vδ2>non-Vδ1Vδ2	5.74	0.0200
**CCL5**	Vδ1>non-Vδ1Vδ2	1.85	0.00879	CD58	Vδ2>non-Vδ1Vδ2	1.45	0.0210
**HLA-B**	Vδ2>non-Vδ1Vδ2	1.32	0.00905	TNF	Vδ1<non-Vδ1Vδ2	0.25	0.0210
**CXCR4**	Vδ2>non-Vδ1Vδ2	1.52	0.0111	CXCL2	Vδ1<non-Vδ1Vδ2	0.12	0.0211
**HLA-A**	Vδ2>non-Vδ1Vδ2	1.29	0.0120	CD59	Vδ1<non-Vδ1Vδ2	0.38	0.0236
**GZMB**	Vδ2>non-Vδ1Vδ2	2.69	0.0169	CD3D	Vδ2>non-Vδ1Vδ2	1.43	0.0238
**IL8**	Vδ1<non-Vδ1Vδ2	0.18	0.0197	FCGRT	Vδ1<non-Vδ1Vδ2	0.16	0.0244
**IL2RG**	Vδ2>non-Vδ1Vδ2	1.43	0.0265	IFNAR2	Vδ2<non-Vδ1Vδ2	0.60	0.0250
**HLA-DRB3**	Vδ1<non-Vδ1Vδ2	0.25	0.0277	TGFB1	Vδ2>non-Vδ1Vδ2	1.43	0.0250
**FYN**	Vδ2>non-Vδ1Vδ2	1.34	0.0288	CD8A	Vδ2<non-Vδ1Vδ2	0.18	0.0256
**NFKBIA**	Vδ1<non-Vδ1Vδ	0.56	0.0323	CD247	Vδ2>non-Vδ1Vδ2<br>Vδ1>non-Vδ1Vδ2	1.55<br>1.53	0.0258<br>0.0300
**CD74**	Vδ1<non-Vδ1Vδ2	0.33	0.0330	CD244	Vδ1>non-Vδ1Vδ2	2.48	0.0260
**PTPRC_all**	Vδ2>non-Vδ1Vδ2	1.35	0.0405	TBX21	Vδ2>non-Vδ1Vδ2	2.22	0.0261
**IL2RB**	Vδ1>non-Vδ1Vδ2	1.60	0.0433	LILRB1	Vδ2<non-Vδ1Vδ2	0.12	0.0269
				IKZF2	Vδ2<non-Vδ1Vδ2	0.24	0.0278
				CEBPB	Vδ1<non-Vδ1Vδ2	0.41	0.0282
				NOTCH2	Vδ1<non-Vδ1Vδ2	0.50	0.0285
**KLRG1**	Vδ2>non-Vδ1Vδ2	3.94	0.000175	APP	Vδ1<non-Vδ1Vδ2	0.14	0.0293
**ZBTB16**	Vδ2>non-Vδ1Vδ2	9.65	0.000629	CCR7	Vδ1<non-Vδ1Vδ2	0.38	0.0297
**GZMK**	Vδ2>non-Vδ1Vδ	4.89	0.000892	CXCL13	Vδ2<non-Vδ1Vδ2	0.03	0.0300
**CCR5**	Vδ2>non-Vδ1Vδ2	2.46	0.00120	IL10RA	Vδ1>non-Vδ1Vδ2	1.28	0.0301
**CXCR3**	Vδ2>non-Vδ1Vδ2	2.27	0.00159	IRF1	Vδ1<non-Vδ1Vδ2	0.63	0.0320
**IL12RB1**	Vδ2>non-Vδ1Vδ2	2.45	0.00211	KLRC2	Vδ2>non-Vδ1Vδ2	3.41	0.0320
**NFATC2**	Vδ2>non-Vδ1Vδ2	1.71	0.00211	CD45RB	Vδ2>non-Vδ1Vδ2	1.58	0.0328
**KLRC1**	Vδ2>non-Vδ1Vδ2	7.26	0.00253	KIR_inhinitin<br>g_Subgroup_2	Vδ1>non-Vδ1Vδ2	4.26	0.0331
**KLRB1**	Vδ2>non-Vδ1Vδ2	3.05	0.00263	CD3E	Vδ2>non-Vδ1Vδ2	1.36	0.0345
**TIGIT**	Vδ2<non-Vδ1Vδ2	0.25	0.00275	LAIR1	Vδ1<non-Vδ1Vδ2	0.27	0.0347
**SH2D1A**	Vδ2>non-Vδ1Vδ2	1.76	0.00354	PIGR	Vδ1<non-Vδ1Vδ2	0.09	0.0361
**STAT4**	Vδ2>non-Vδ1Vδ2	2.11	0.00359	CXCL1	Vδ1<non-Vδ1Vδ2	0.14	0.0375
**TGFBI**	Vδ1<non-Vδ1Vδ2	0.04	0.00367	CTSS	Vδ1<non-Vδ1Vδ2	0.64	0.0384
**LAG3**	Vδ2>non-Vδ1Vδ2	2.20	0.00474	TNFRSF14	Vδ1<non-Vδ1Vδ2	0.89	0.0387
**IFNGR1**	Vδ2>non-Vδ1Vδ2	2.04	0.00504	RARRES3	Vδ1>non-Vδ1Vδ2	1.43	0.0404
**PTGER4**	Vδ2>non-Vδ1Vδ2	1.63	0.00647	MX1	Vδ1<non-Vδ1Vδ2	0.71	0.0442
**LGALS3**	Vδ1<non-Vδ1Vδ2	0.33	0.00709	ITGAX	Vδ2<non-Vδ1Vδ2	0.23	0.0453
**SMAD3**	Vδ2>non-Vδ1Vδ2	2.20	0.00812	CTNNB1	Vδ2<non-Vδ1Vδ2	0.72	0.0474
**HLA-DQA1**	Vδ1<non-Vδ1Vδ2	0.16	0.00935	KIR_inhinitin<br>g_Subgroup_1	Vδ1>non-Vδ1Vδ2	3,39	0.0483
				LEF1	Vδ2<non-Vδ1Vδ2	0.19	0.0494

*p-values were calculated using two-tailed Friedman test and not adjusted for multiple comparisons

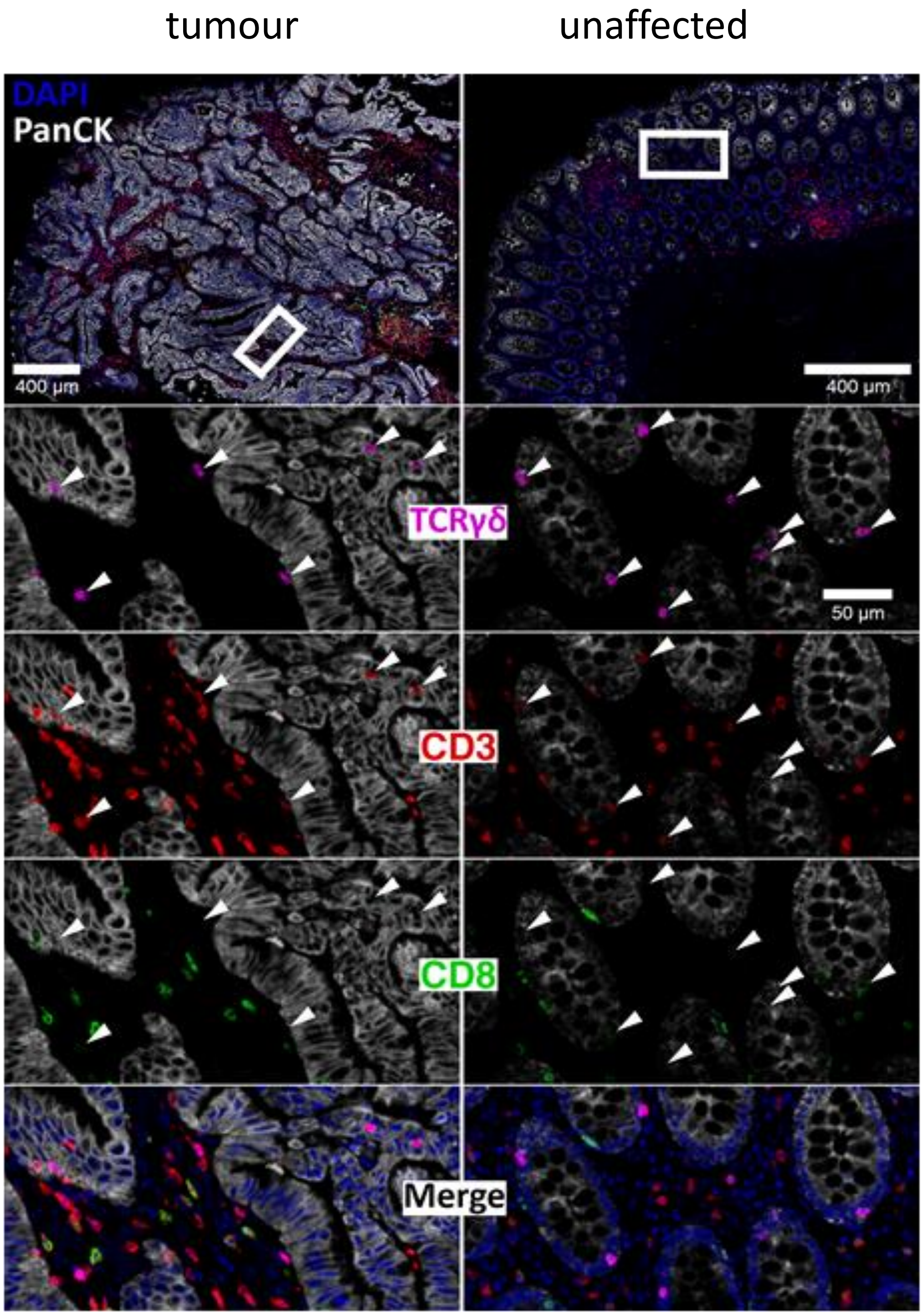

Suppl. Fig. S1 Immunflourescence images showing staining for cytokeratin (panCK; white), TCRγδ (purple), CD3 (red), CD8 (green) and DAPI (blue) in a colon tumor and the unaffected colon mucosa from the same patient.

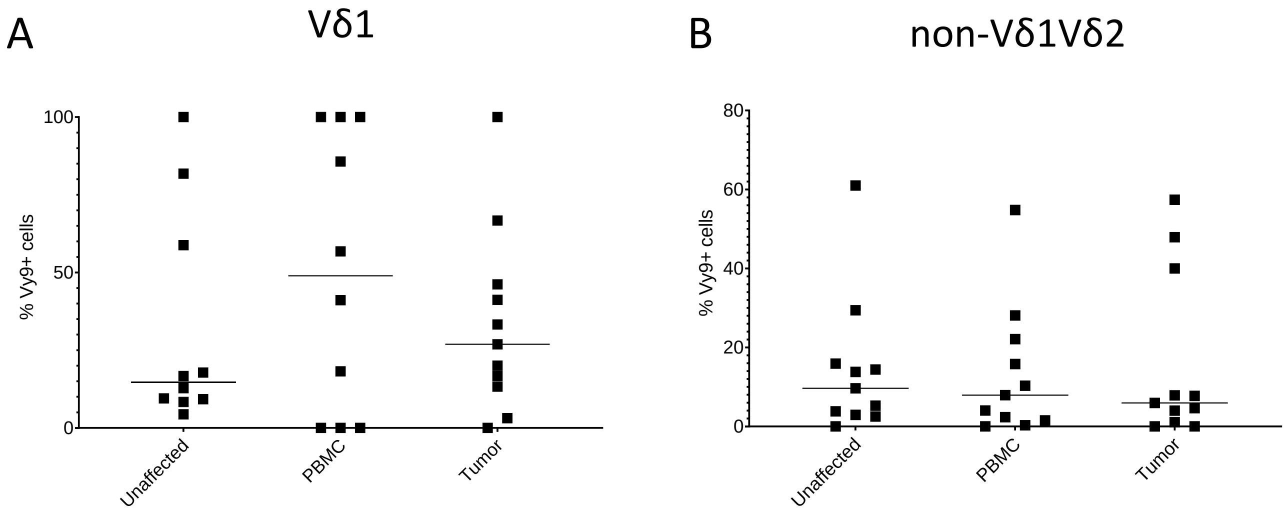

Suppl. Fig. S2 Vγ9 expression in tumour-infiltrating γδ T cells. Single cell suspensions were isolated from tumours, corresponding unaffected colon mucosa, and blood, and Vδ1 (A) and non-Vδ1Vδ2 (B) γδ T cells were analysed for the expression of Vy9. Symbols represent individual values and the line the median. n=11.

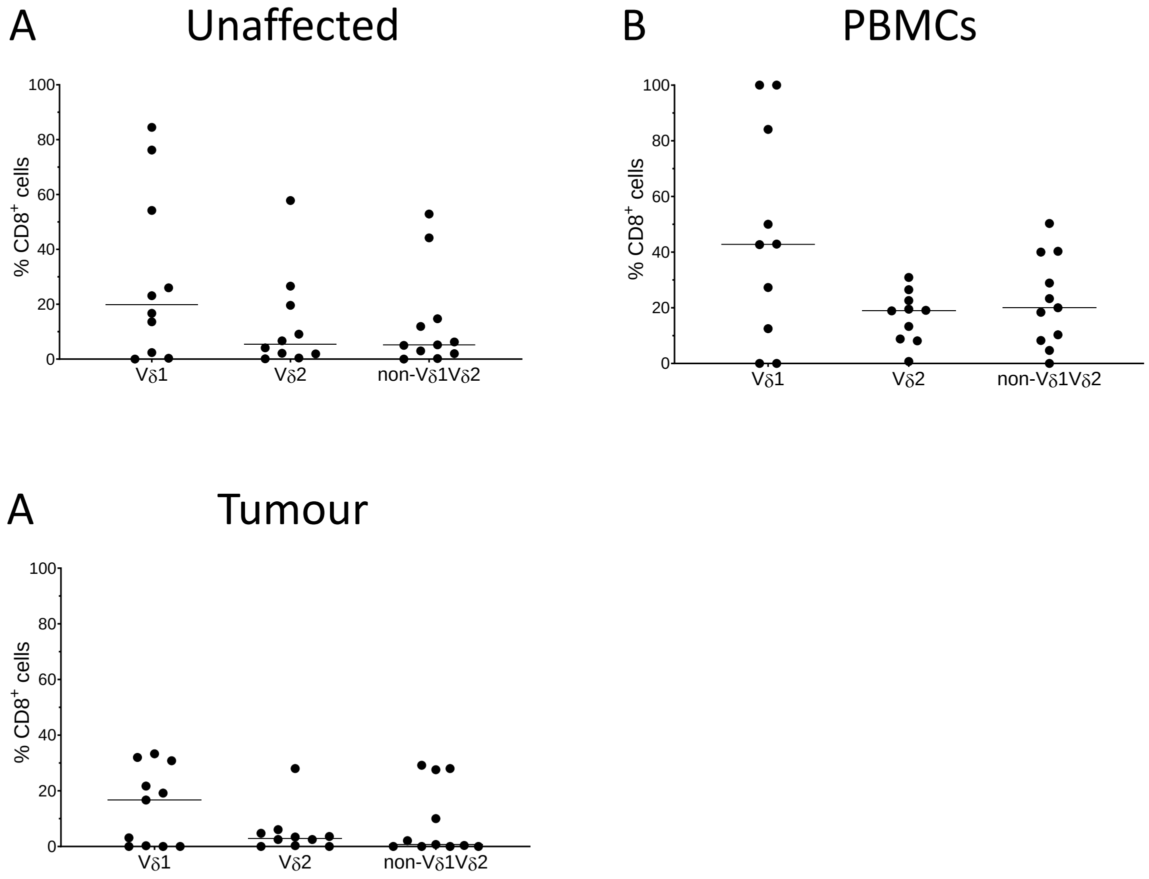

Suppl. Fig. S3 CD8 expression in tumour-infiltrating γδ T cells. Single cell suspensions were isolated from tumours, corresponding unaffected colon mucosa, and blood, and γδ T cells were analysed for their expression of CD8. Graphs show the expression of CD8 among γδ T cells from the unaffected mucosa (A), blood (B), and tumours (C) Symbols represent individual values and the line the median. n=11.

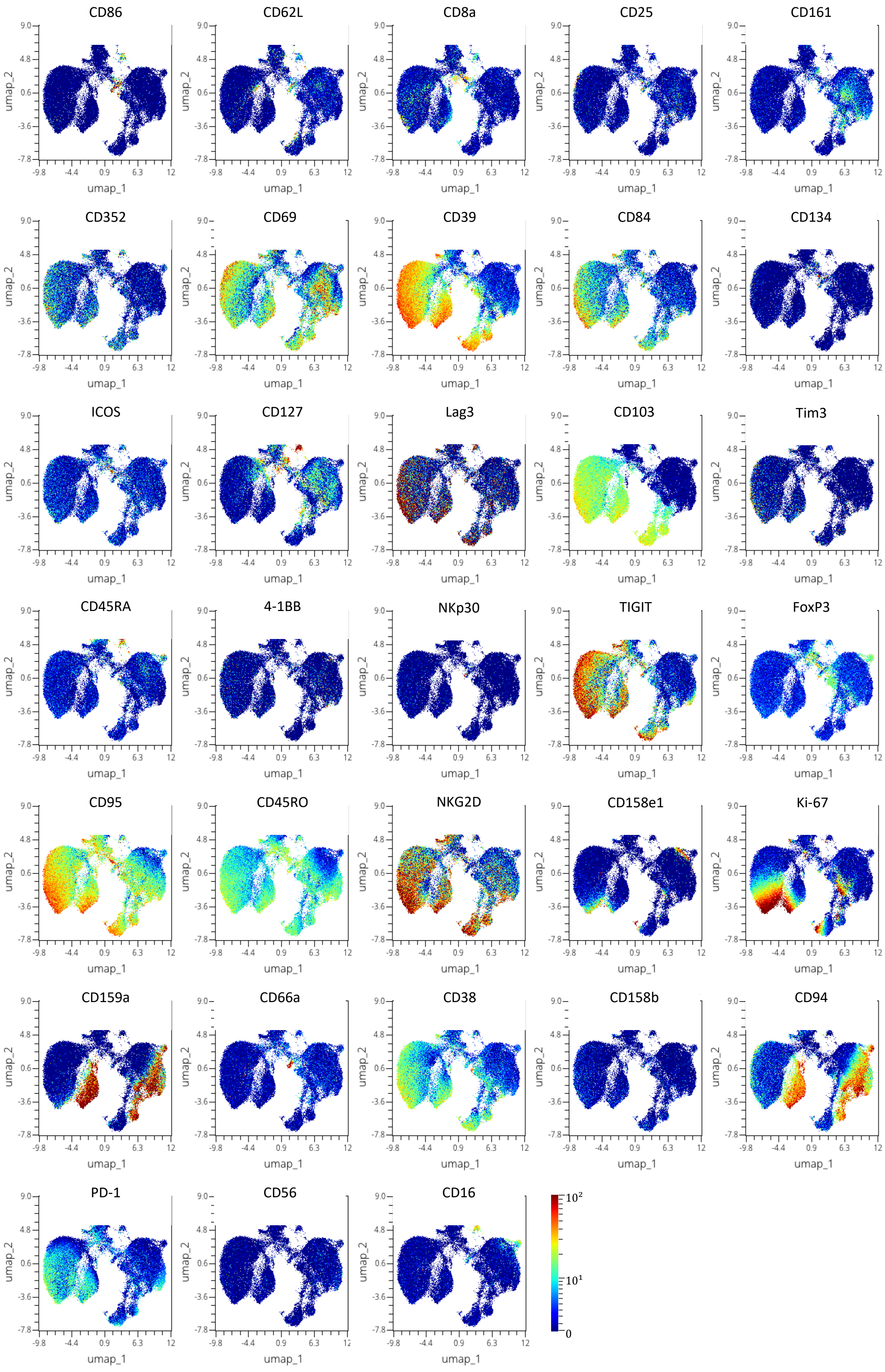

Suppl. Fig. S4 Clustering analysis of tumour-infiltrating γδ T cells. Single cell suspensions were isolated from tumours and analysed using mass cytometry. Expression of each marker included in the analysis is overlaid separately on the UMAP/Phenograph plot. The colour scale represents staining intensity of the respective markers, scaled based on the highest signal in each specific marker. n=18

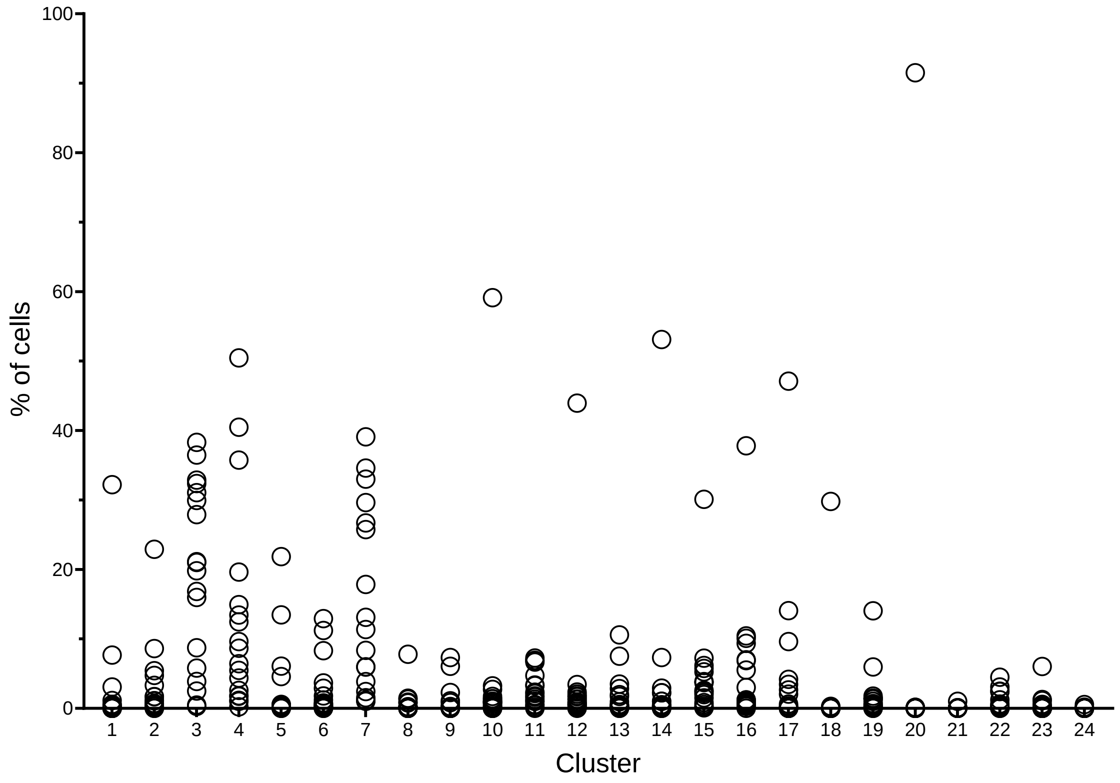

Suppl. Fig. S5 Distribution of γδ T cells from individual tumours between the UMAP clusters. Symbols show the percentage of γδ T cells from an individual tumour that ended up in a certain cluster. n=18

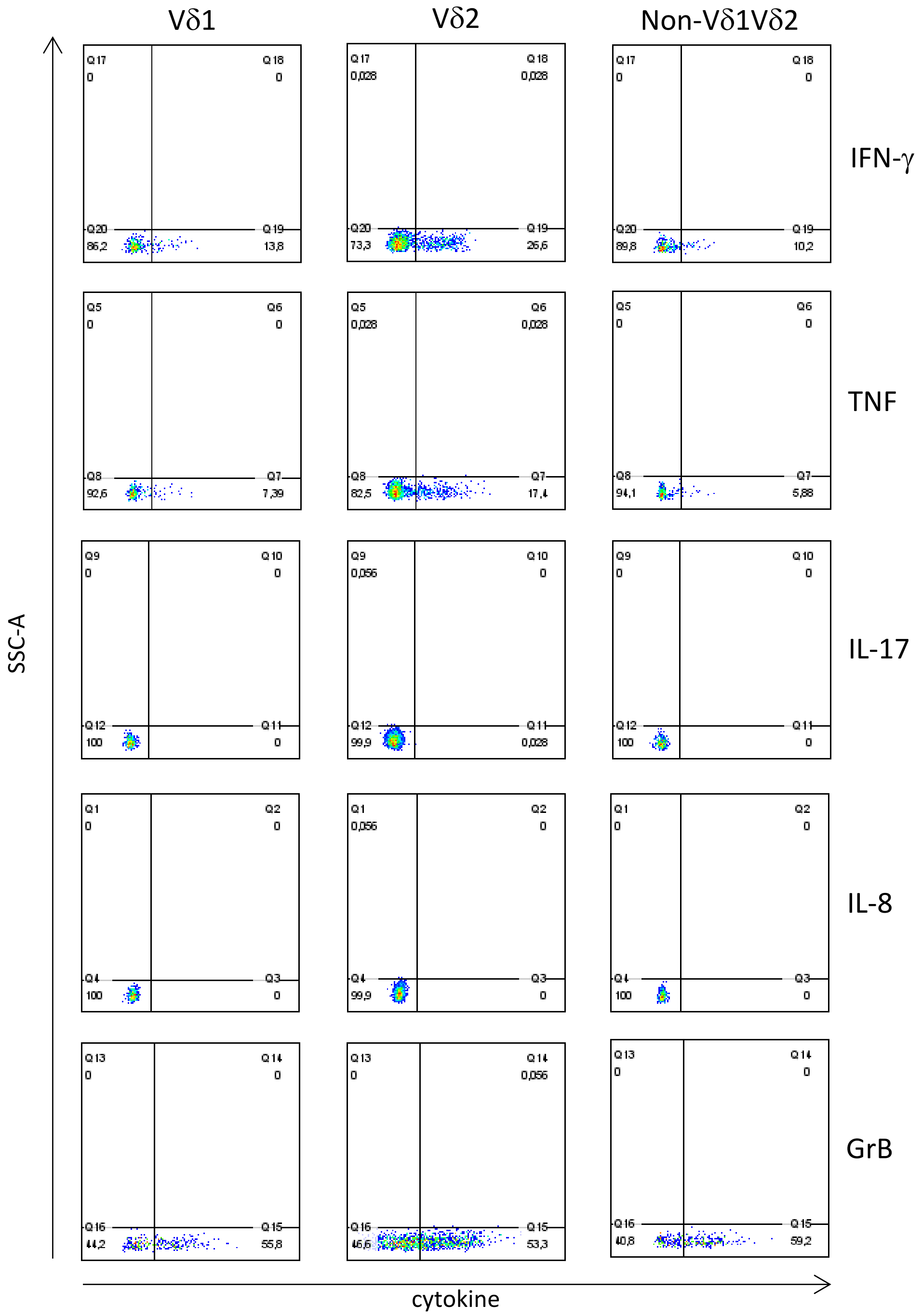

Suppl. Fig. S6. Cytokines and effector proteins in tumor-infiltrating γδ T cells. Single cell suspensions were isolated from a colon adenocarcinoma and stimulated with PMA and Ionomycin. Vδ1, Vδ2, and non-Vδ1Vδ2 cells were analysed for the expression of IFN-γ, TNF, IL-17A, IL-8, and GrB by flow cytometry. Dot-plots from one representative patient (the same patient as in Suppl. Fig. S7 and S8) is shown.

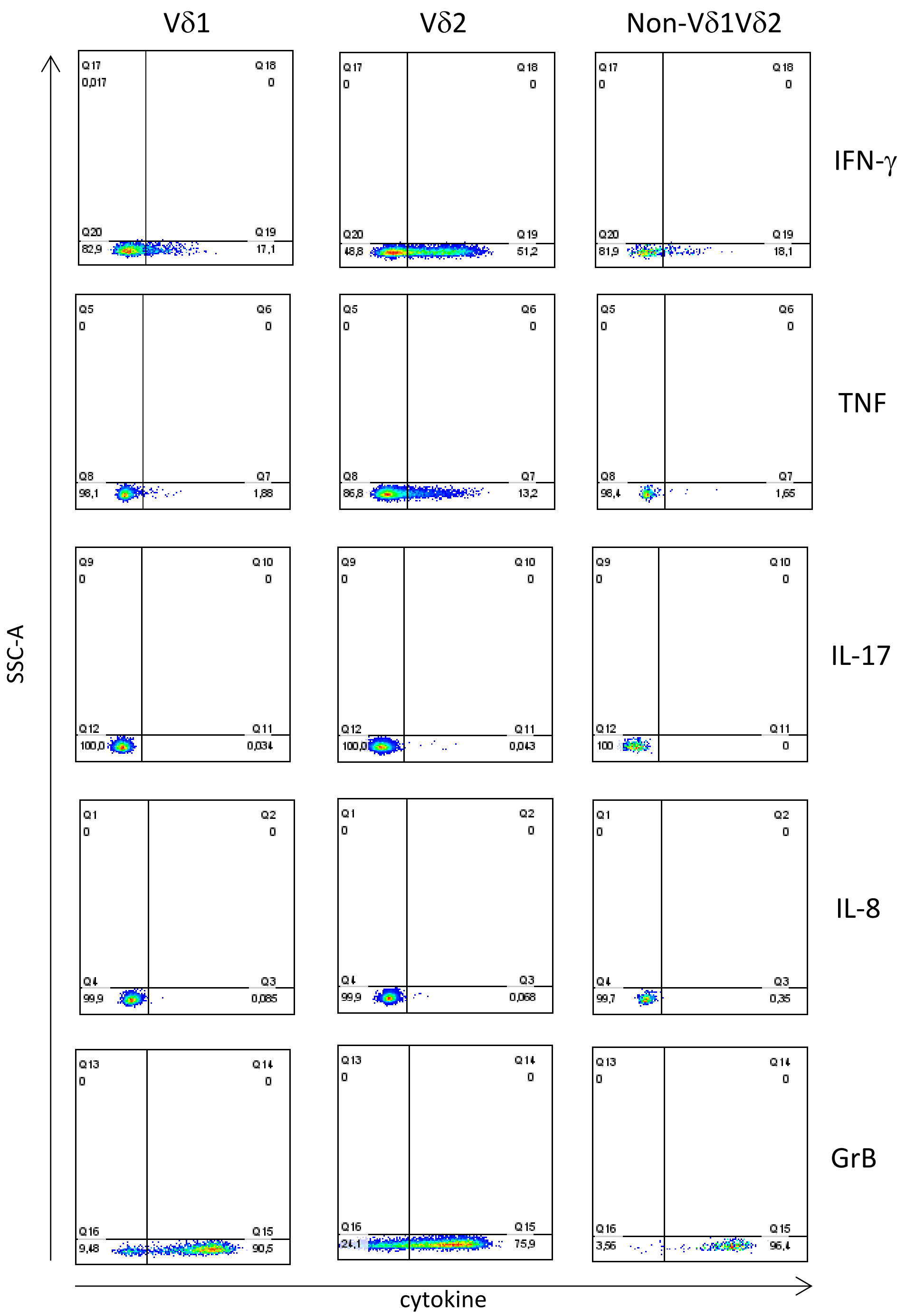

Suppl. Fig. S7. Cytokines and effector proteins in circulating γδ T cells. Single cell suspensions were isolated from blood and stimulated with PMA and Ionomycin. Vδ1, Vδ2, and non-Vδ1Vδ2 cells were analysed for the expression of IFN-γ, TNF, IL-17A, IL-8, and GrB by flow cytometry. Dot-plots from one representative patient (the same patient as in Suppl. Fig. S6 and S8) is shown.

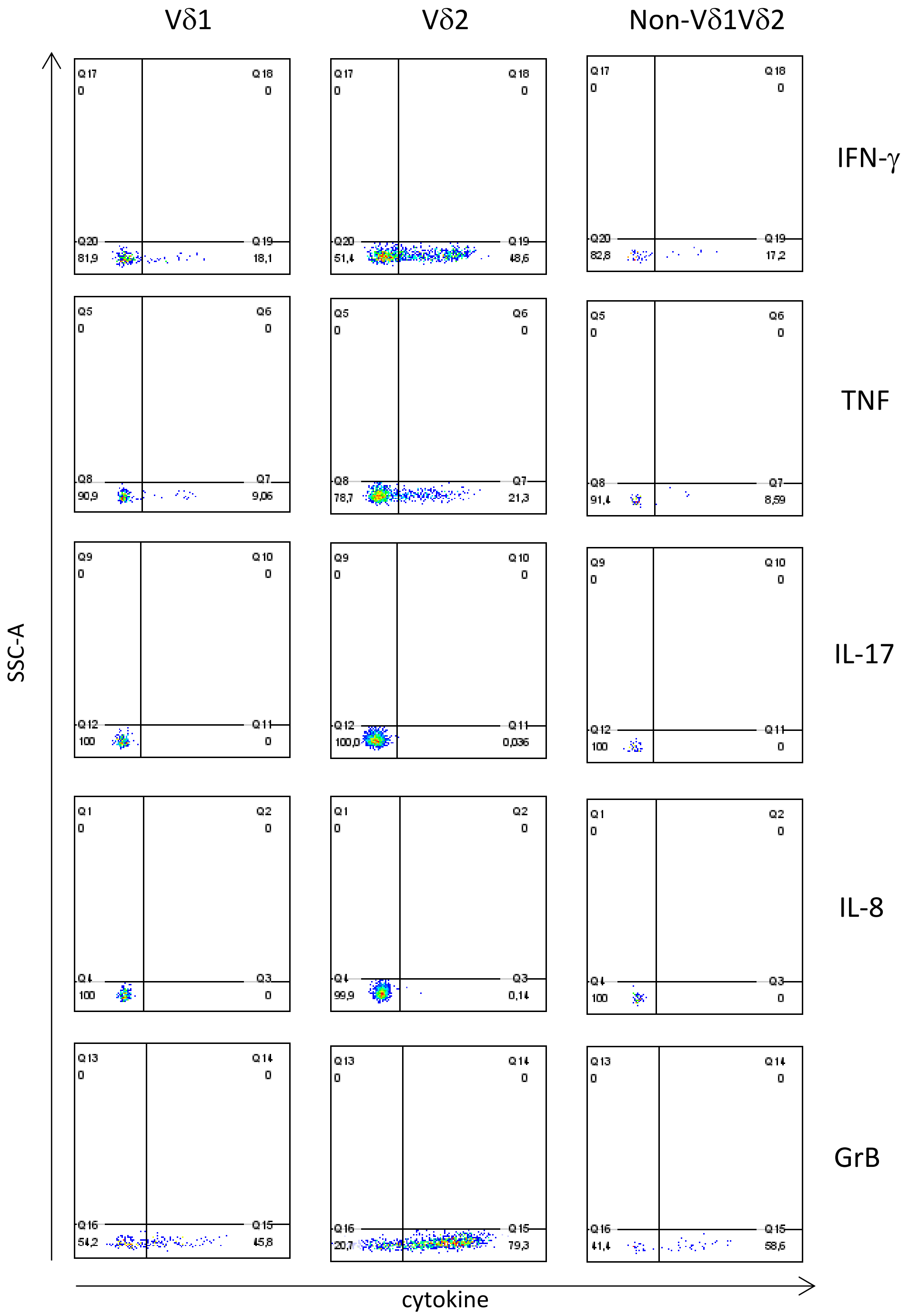

Suppl. Fig. S8. Cytokines and effector proteins in colon γδ T cells. Single cell suspensions were isolated from unaffected colon tissue and stimulated with PMA and Ionomycin. Vδ1, Vδ2, and non-Vδ1Vδ2 cells were analysed for the expression of IFN-γ, TNF, IL-17A, IL-8, and GrB by flow cytometry. Dot-plots from one representative patient (the same patient as in Suppl. Fig. S6 and S7) is shown.

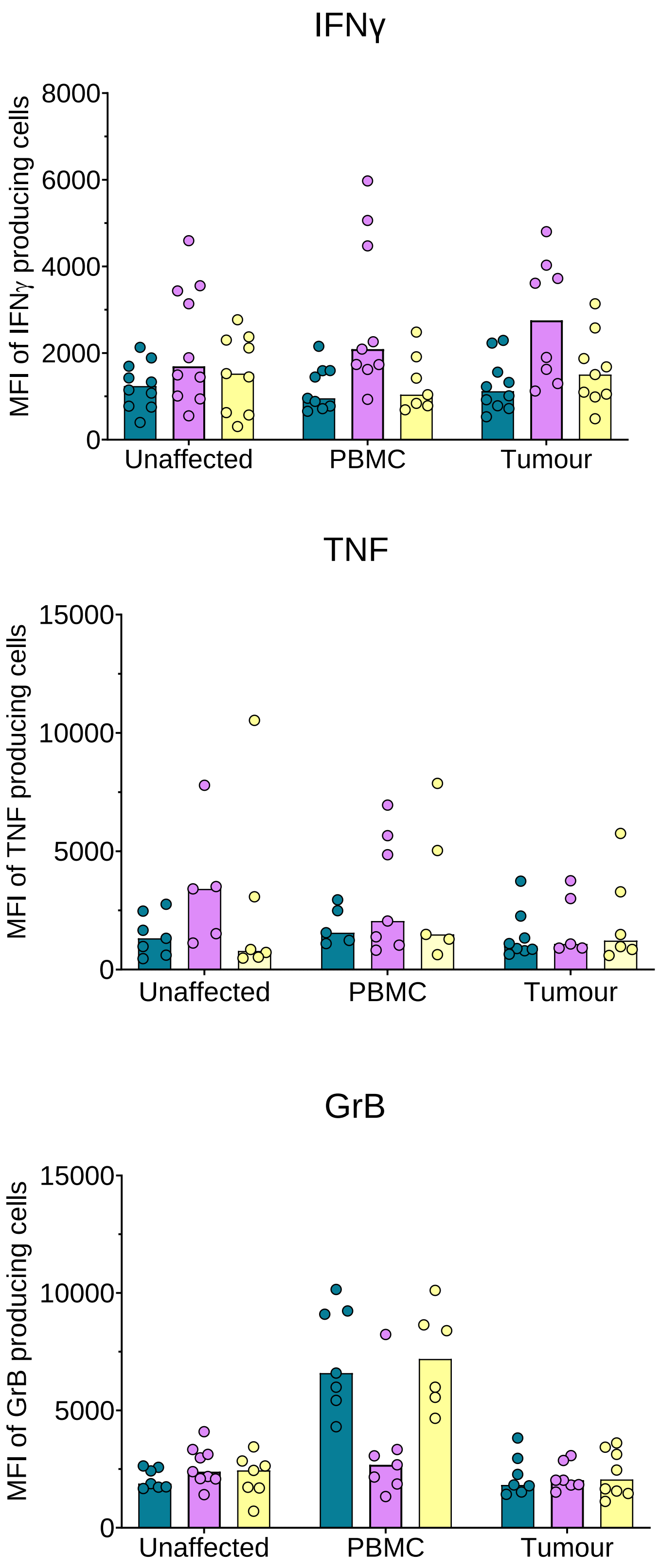

Suppl. Fig. S9. Cytokines and effector proteins in γδ T cells. Single cell suspensions were isolated from blood, unaffected colon, and tumor tissue and stimulated with PMA and Ionomycin. Vδ1, Vδ2, and non-Vδ1Vδ2 cells were analysed for the expression of IFN-γ, TNF, and GrB by flow cytometry and the median fluorescence intensity (MFI) determined for the cytokine-producing cells. Symbols represent individual values and the bars the median. n=5-10

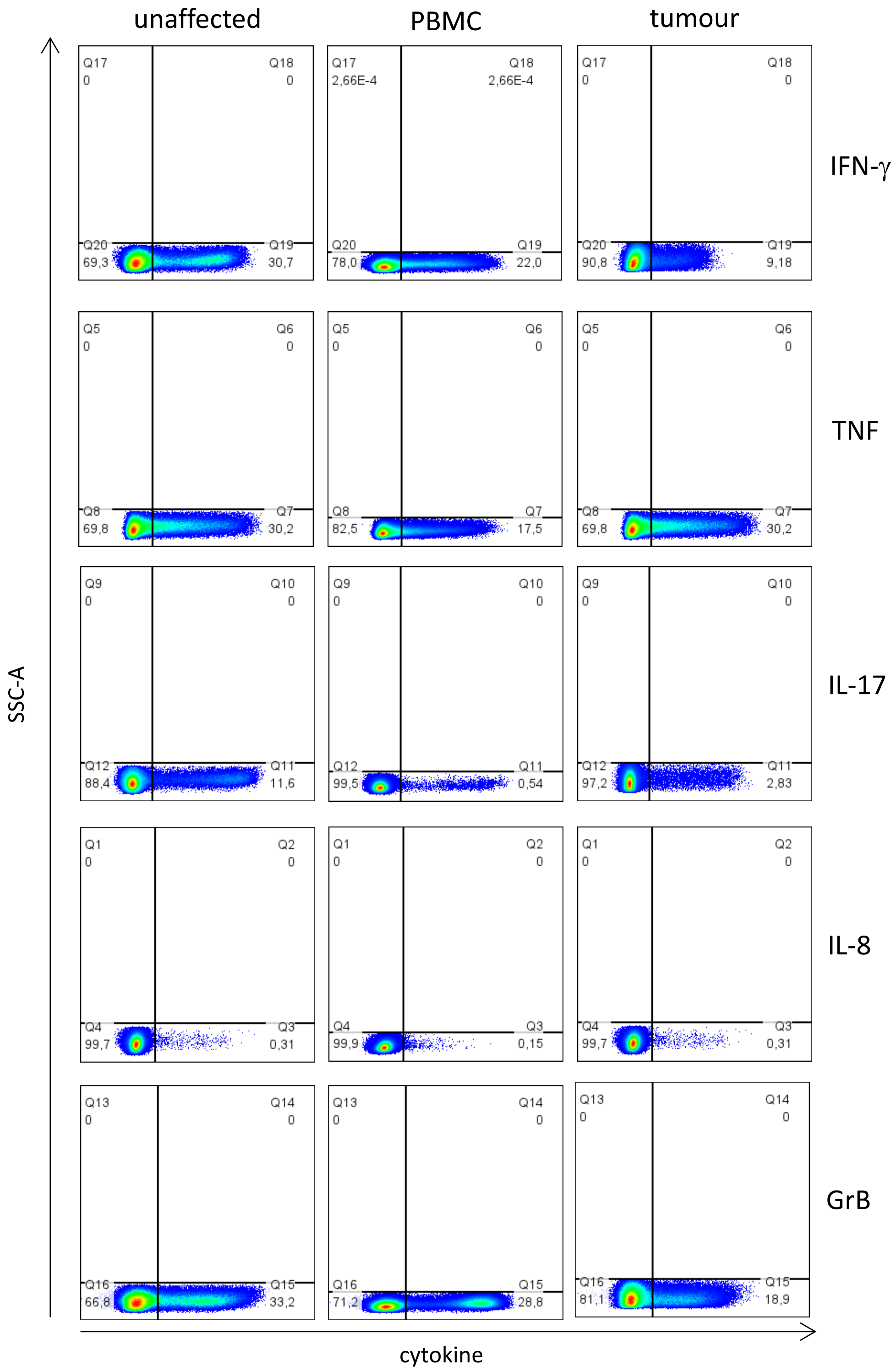

Suppl. Fig. S10. Cytokines and effector proteins in αβ T cells. Single cell suspensions were isolated from blood, unaffected colon tissues, and tumours, stimulated with PMA and Ionomycin, and analysed for the expression of IFN-γ, TNF, IL-17A, IL-8, and GrB by flow cytometry. Dot-plots from one representative patient (the same patient as in Suppl. Fig. S6-S8) is shown.
